# Semantic representation of reported measurements in radiology

**DOI:** 10.1186/s12911-016-0248-9

**Published:** 2016-01-22

**Authors:** Heiner Oberkampf, Sonja Zillner, James A. Overton, Bernhard Bauer, Alexander Cavallaro, Michael Uder, Matthias Hammon

**Affiliations:** 1 Department of Computer Science, Software Methodologies for Distributed Systems, University of Augsburg, Universitätsstraße 6a, 86159 Augsburg, Germany; 2 Corporate Technology, Siemens AG, Otto-Hahn-Ring 6, 81739 Münech, Germany; 3 School of International Business and Entrepreneurship, Steinbeis University, Kalkofenstraße 53, 71083 Herrenberg, Germany; 4 Knocean.com, Toronto, Canada; 5 Department of Radiology, University Hospital Erlangen, Maximiliansplatz 1, 91054 Erlangen, Germany

**Keywords:** Radiology, Measurement, Classification, Ontology, OBO, Open Biological and Biomedical Ontologies, RECIST, Follow-up

## Abstract

**Background:**

In radiology, a vast amount of diverse data is generated, and unstructured reporting is standard. Hence, much useful information is trapped in free-text form, and often lost in translation and transmission. One relevant source of free-text data consists of reports covering the assessment of changes in tumor burden, which are needed for the evaluation of cancer treatment success. Any change of lesion size is a critical factor in follow-up examinations. It is difficult to retrieve specific information from unstructured reports and to compare them over time. Therefore, a prototype was implemented that demonstrates the structured representation of findings, allowing selective review in consecutive examinations and thus more efficient comparison over time.

**Methods:**

We developed a semantic Model for Clinical Information (MCI) based on existing ontologies from the Open Biological and Biomedical Ontologies (OBO) library. MCI is used for the integrated representation of measured image findings and medical knowledge about the normal size of anatomical entities. An integrated view of the radiology findings is realized by a prototype implementation of a ReportViewer. Further, RECIST (Response Evaluation Criteria In Solid Tumors) guidelines are implemented by SPARQL queries on MCI. The evaluation is based on two data sets of German radiology reports: An oncologic data set consisting of 2584 reports on 377 lymphoma patients and a mixed data set consisting of 6007 reports on diverse medical and surgical patients. All measurement findings were automatically classified as abnormal/normal using formalized medical background knowledge, i.e., knowledge that has been encoded into an ontology. A radiologist evaluated 813 classifications as correct or incorrect. All unclassified findings were evaluated as incorrect.

**Results:**

The proposed approach allows the automatic classification of findings with an accuracy of 96.4 % for oncologic reports and 92.9 % for mixed reports. The ReportViewer permits efficient comparison of measured findings from consecutive examinations. The implementation of RECIST guidelines with SPARQL enhances the quality of the selection and comparison of target lesions as well as the corresponding treatment response evaluation.

**Conclusions:**

The developed MCI enables an accurate integrated representation of reported measurements and medical knowledge. Thus, measurements can be automatically classified and integrated in different decision processes. The structured representation is suitable for improved integration of clinical findings during decision-making. The proposed ReportViewer provides a longitudinal overview of the measurements.

## Background

In radiology, a vast amount of diverse data is generated (image data, secondary captures, texts, etc.). Obviously, these data facilitate the generation of fountains of knowledge and pools of evidence supporting decision-making as well as therapy planning and monitoring. Unfortunately, because unstructured reporting is the norm, much useful information is trapped in free-text form, and often lost in translation and transmission [1, 2].

The assessment of changes in tumor burden is an important task during the evaluation of cancer treatment success. Tumor shrinkage and disease progression must be assessed and reported to evaluate therapy response. Increasingly many different measurements are performed and reported in radiology (volumes, perfusion or diffusion measurements, spectroscopy results, etc.). However, most of these data are measurements of the size of a lesion or an organ in different dimensions. The change of the lesion size is still the critical factor in follow-up examinations (sonography, computed tomography, magnetic resonance imaging) during therapy or surveillance. The RECIST (Response Evaluation Criteria In Solid Tumors) guideline allows a standardized assessment of tumor burden and tumor response during therapy [3]. Here, a sum of the diameters (short axis for lymph nodes, longest diameter for the remaining lesions) for all target lesions is calculated and reported as the baseline or follow-up sum and indicates therapy response/failure [3].

In general, the increase in available clinical data and availability of medical knowledge provide the basis for better and more effective decision-making. A common assumption is that the more data we have about a particular patient, the more effectively this patient can be treated. However, this requires the automatic and longitudinal integration of findings from different reports in decision processes such as diagnosis or treatment evaluation. Today, however, improved quality of treatment on the one hand and limited funding on the other hand are in conflict. In the current situation, the availability of more data entails increased efforts for the clinicians and thus higher costs. This is mostly due to three challenges:

1. Only a small amount of the data is structured, while a high percentage of relevant data is unstructured.
2. Existing data are not sufficiently linked to medical knowledge and thus do not include the appropriate level of detail for decision-support systems.
3. Finding descriptions are not longitudinally integrated. Thus, the comparison of findings from consecutive examinations to evaluate the change of the health status requires extensive manual effort.

**Fig. 1:**
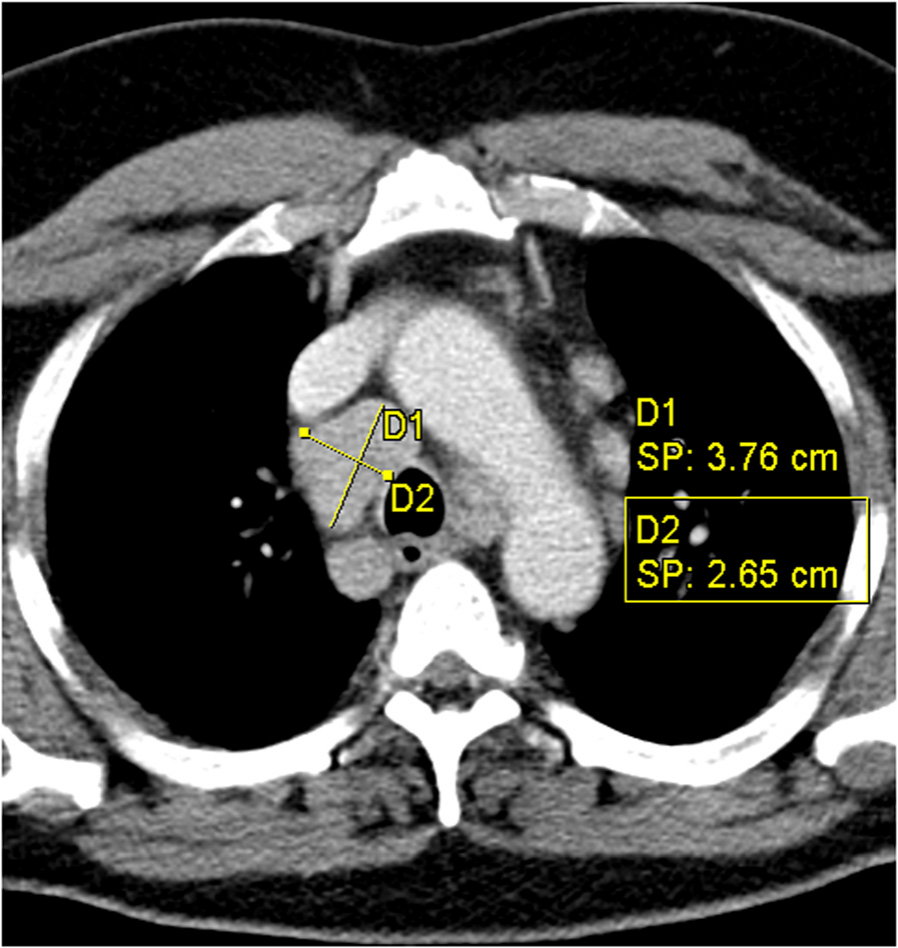
Example of an abnormal mediastinal lymph node. An axial image of a contrast-enhanced computed tomography examination of the thorax. The size of a lymph node is specified by measuring the longest in-plane diameter and the corresponding short axis in the orthogonal direction

As a result of these problems, most of the available data are simply not used to their full potential during clinical decision-making. To overcome these problems, structured representations of findings are needed that allow the selective retrieval and better integration of useful data. For instance, during diagnosis and examination processes, attention is mainly focused on abnormalities. For blood tests, a classification of measurement values (low, normal, high) is commonly provided. However, for image findings, this kind of automatic normality classification is missing, since radiology findings are more complex (see, e.g., Fig. 1).

Much work has already been performed on developing structured representations for clinical findings; this is commonly realized through a combination of an information model with reference terminologies [4–6]. The role of the information model is to define the schema according to which the terminology is used. In previous work [7], we created a corresponding Model for Clinical Information (MCI) that is based on ontologies from the Open Biological and Biomedical Ontologies (OBO) library [8, 9]. RadLex [10], the Foundational Model of Anatomy [11], and other ontologies are employed as reference terminologies. Further, in [12], we demonstrated how structured representations of measurement findings can be extracted from free-text radiology reports. The contribution of this paper is twofold:

1. We describe classes and properties of MCI that are used for the structured representation of size measurements typically found in radiology reports and medical knowledge about the normal size of anatomical entities.
2. We show how measurement information is enriched using formalized medical background knowledge in order to provide different views on the data required by radiologists and referring physicians for more efficient decision-making. For instance, we classify findings as normal or abnormal and identify possible target lesions according to RECIST guidelines.

In a prototype implementation (ReportViewer), we demonstrate the usage of our structured representations. The ReportViewer allows selective review of reported findings from consecutive examinations and thus more efficient comparison of radiological findings over time. Before we conclude, the classification algorithm is evaluated and related work is discussed.

## Methods

In this section, we describe the Model for Clinical Information (MCI), focusing on structured representations of measured image findings. Then we describe the knowledge model with normal size specifications of anatomical entities and how it is used to classify image findings as well as integrating RECIST guidelines. Finally, the ReportViewer prototype is presented.

This single-center investigation was approved by the institutional review board of the University Hospital Erlangen, and all procedures were in accordance with the Helsinki Declaration. The need for informed consent was waived.

### The model for clinical information

As described in [7], the Model for Clinical Information (MCI) is based on selected upper- and mid-level ontologies from the Open Biological and Biomedical Ontologies (OBO) Foundry. The OBO Foundry follows the idea of ‘coordinated evolution of orthogonal ontologies’ that are based on a common architecture to support biomedical data integration [8]. The common architecture necessary for this alignment is provided by a common upper-level ontology - namely the Basic Formal Ontology (BFO) [13]. Additionally, the OBO Foundry has developed naming conventions described in [14] that support the coordinated creation of ontologies in a common framework. MCI follows the OBO naming conventions and reuses all classes of BFO and other selected classes and properties from the Relations Ontology (RO) [15], the Ontology for General Medical Science (OGMS) [16], the Information Artifact Ontology (IAO) [17], the Ontology for Biomedical Investigations (OBI) [18], the Phenotypic Quality Ontology (PATO) [19], the Units Ontology (UO) [20], and the Foundational Model of Anatomy (FMA) [11]. These ontologies define the basic classes and properties that provide the foundation of our model, and already provide a basic structure for the representation of clinical findings. In total, MCI contains 551 classes (447 imported), 107 object properties (83 imported), and 33 data properties (15 imported).

#### Basic representation of clinical findings

**Fig. 2:**
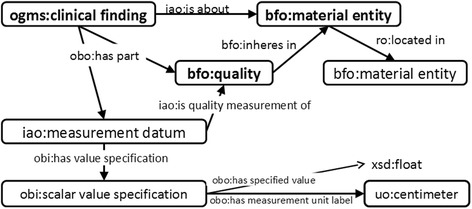
The basic pattern of a clinical finding. A clinical finding always describes a quality of some material entity. Additionally, the quality can be a described by a measurement, and the location of the described entity can be specified

**Fig. 3:**
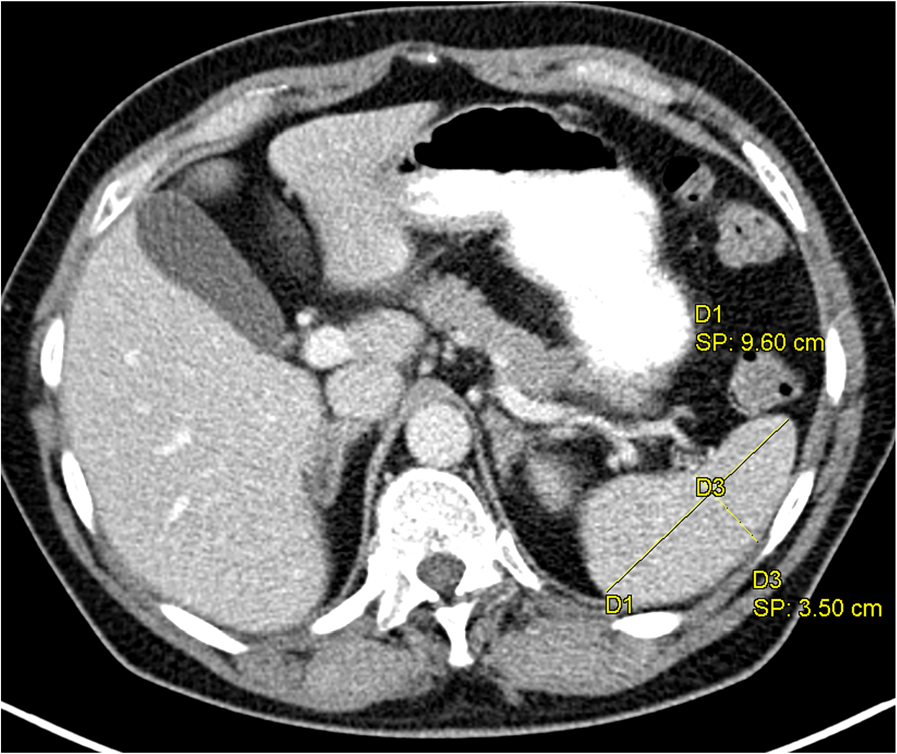
Example of a normal spleen. An axial image of a contrast-enhanced computed tomography examination of the upper abdomen. The size of the spleen in the axial plane is specified by measuring the length and width at the hilum

OGMS defines a clinical finding as “a representation that is either the output of a clinical history taking or a physical examination or an image finding, or some combination thereof” [16]. In its most basic form, a clinical finding describes a quality of some material entity (Fig. 2). Additionally, the quality can be specified by a measurement; e.g., the measured length of the spleen (Fig. 3) can be represented by using this pattern.

#### Extensions for detailed representation of measurement findings

The basic pattern of clinical findings is useful and provides an effective structure for extensions that we developed in order to express image findings at the required level of detail. In the following paragraphs, we describe the extensions (i.e. classes and properties) required for precise representation of specific finding types and the orientation of measurements.

#### Types of clinical findings

**Fig. 4:**
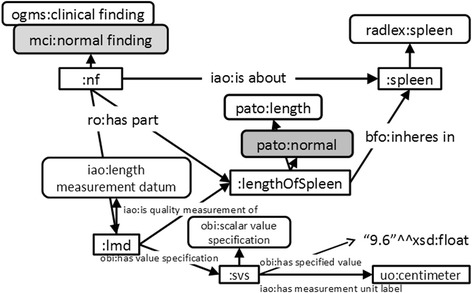
Spleen with normal length. A length measurement finding was represented with OBO classes and relations. Boxes with round corners denote classes, otherwise instanced. Named arrows denote relations between instances. Arrows without labels denote rdf:type relations from individuals to corresponding classes

Clinical findings are central information objects beyond all patient data because they are the main input for decision-making, such as providing a diagnosis or treatment evaluation. For selective retrieval and usage of sought information, MCI defines approximately 30 subclasses of clinical findings [7]. In particular, MCI defines a normal finding as “a clinical finding describing some normal quality that inheres in some anatomical structure.” The class of abnormal findings is defined accordingly and is disjoint with normal findings. The distinction between abnormal and normal findings is important during radiological reading because it affects the management and the prognosis of the patient. Figure 4 shows the representation of a normal finding related to the spleen length from Fig. 3.

#### Orientation of measurements

**Fig. 5:**
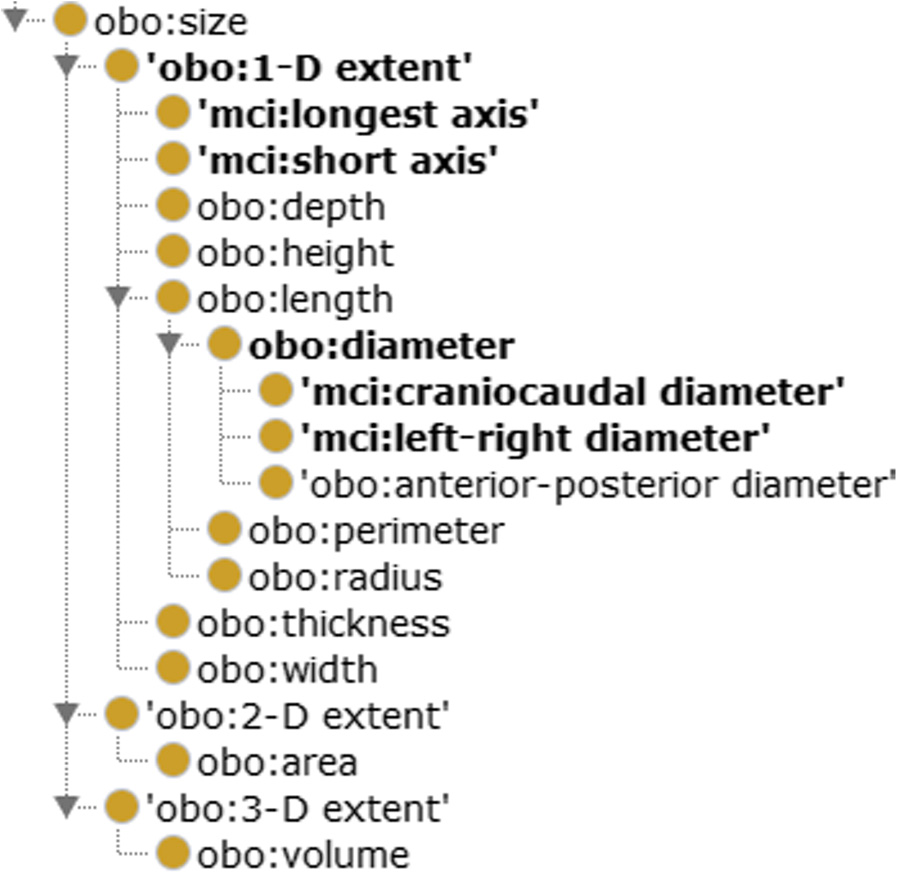
Size qualities. PATO defines several qualities for size. MCI adds four qualities defined as necessary for the representation of image findings

We import several qualities from PATO; however, some extensions needed to be made to capture the orientation aspect of the measured qualities more precisely. As shown in Fig. 5, PATO defines several size qualities, in particular subclasses of 1-D extent such as length, width, height and diameter.

**Fig. 6:**
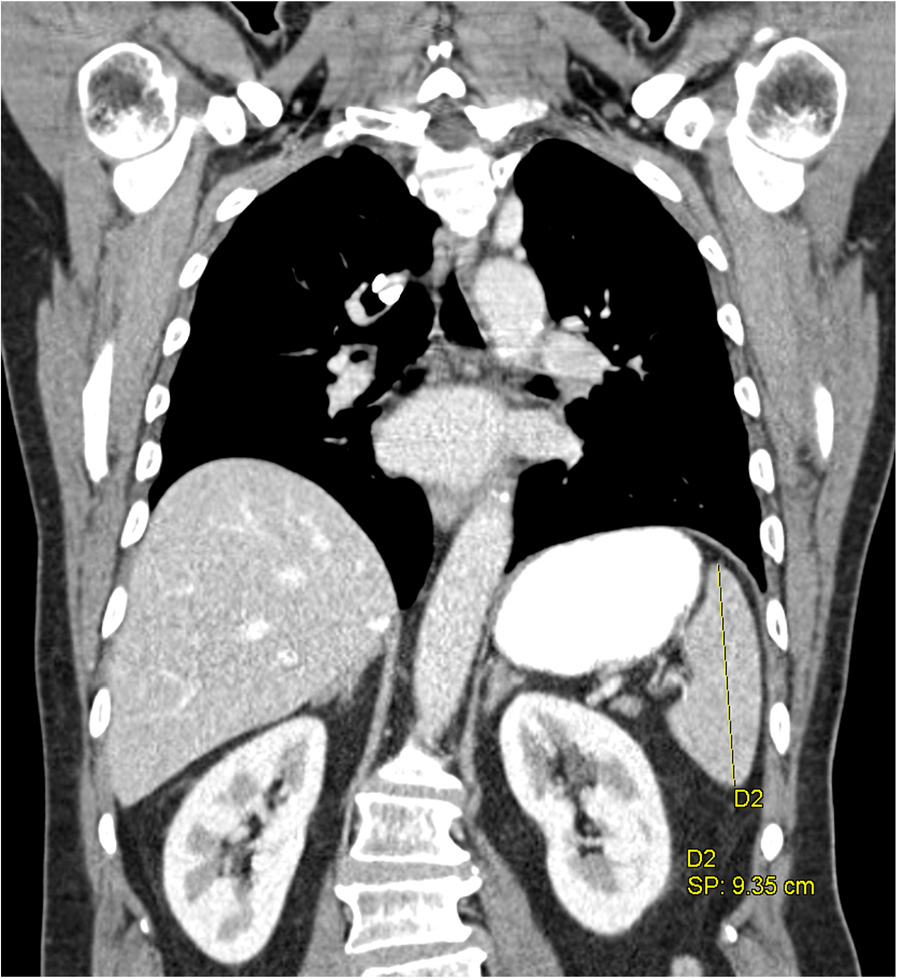
Craniocaudal extension of the spleen. A coronal slice of a contrast-enhanced computed tomography examination of the upper thorax/upper abdomen. The height of the spleen is measured in parallel to the craniocaudal axis

To precisely express image findings, MCI adds the following qualities: the craniocaudal diameter is defined as a diameter that is along the craniocaudal axis, and the left-right diameter is defined as a diameter that is orthogonal to the sagittal plane and parallel to some left-right axis. For instance, the height of the spleen is measured along the craniocaudal axis (as shown in Fig. 6) and thus can be more precisely specified as the craniocaudal diameter of the spleen.

Most of the measurements in radiology are taken in one of the three main body planes (axial or transverse plane, coronal plane, sagittal plane). MCI imports corresponding classes from FMA. To define relations to these planes and axis, we defined the following object properties: “parallel to” is defined as ‘a symmetric property that holds between anatomical planes that do not intersect’ and “orthogonal to” is defined as ‘a symmetric property that holds between anatomical planes that are linearly independent’.

In the description of solid tumors and lymph nodes, the size is specified by the longest 1-D extension and the corresponding longest perpendicular 1-D extension within a specific plane, i.e. the short axis (as shown in Fig. 1). Here, the orientation of the measurement is defined by the shape of the particular anatomical entity (tumor, lymph node, etc.). Accordingly, MCI defines the following two subclasses of 1-D extent: the longest (in plane) axis is ‘a 1-D extent describing the longest straight line, where all points of the line are within the respective object’. The short axis is ‘a 1-D extent describing the longest straight line of an object in orthogonal direction to the longest axis of that object’.

**Fig. 7:**
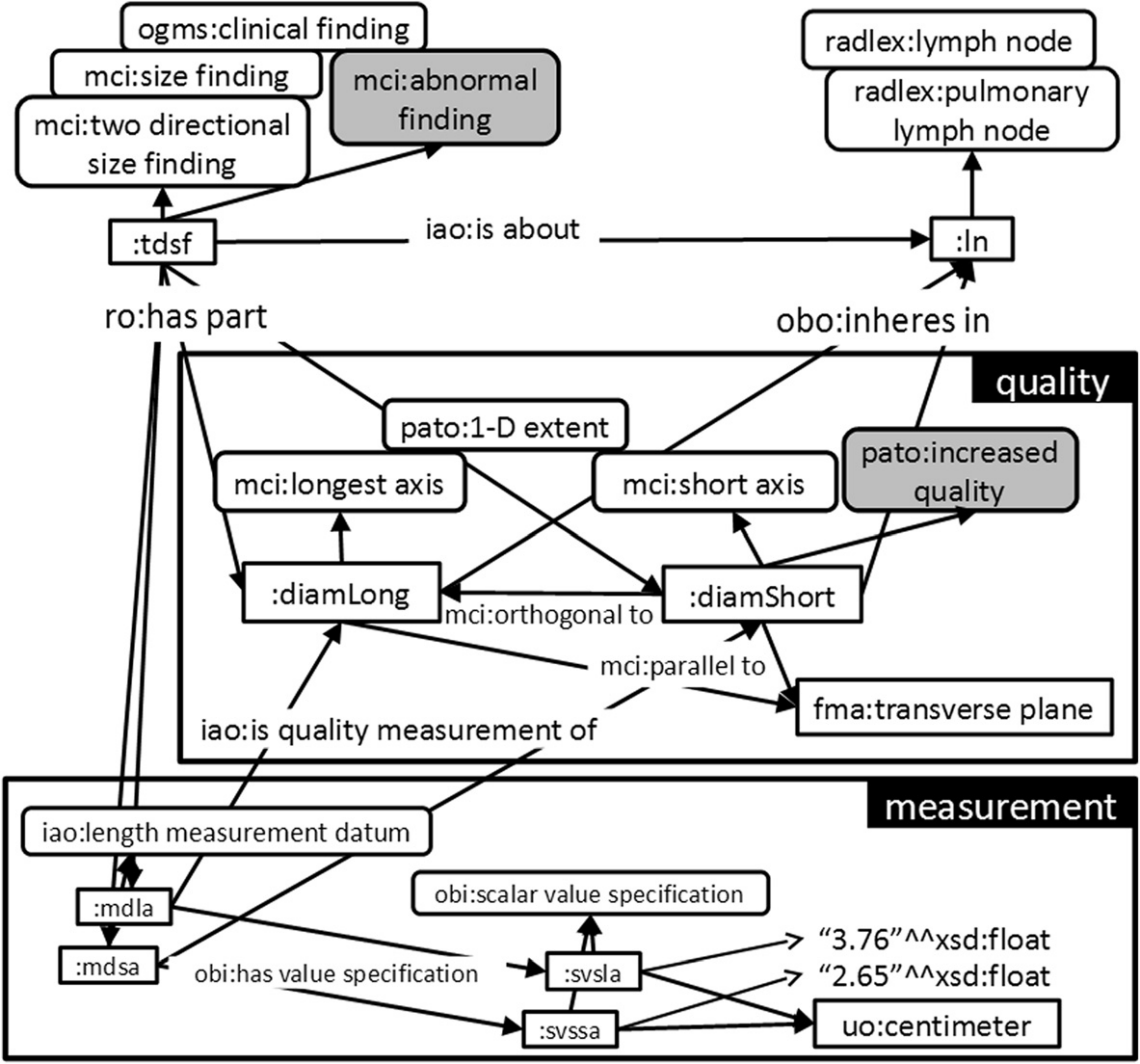
Lymph node size in the axial plane. According to RECIST guidelines, the size of lymph nodes is specified by the longest 1-D extension (longest axis) and the corresponding longest perpendicular 1-D extension (short axis) within the axial plane

Usually, lesions and organs are measured in 3 dimensions. However, due to different reasons, measurements are sometimes performed in 2 or just 1 dimension. Interestingly, during the evaluation according to the RECIST guidelines, only the short axis (lymph nodes) or the longest diameter (remaining lesions) is considered [3]. In other words, the length of the short axis is the relevant quality for classification of malignancy/abnormality [21–24]. The final representation of a RECIST-compliant measurement finding of a lymph node is shown in Fig. 7.

### Knowledge models

Detailed representation of image findings and the distinction between normal and abnormal findings is important, and MCI provides corresponding classes. However, the distinction is often not explicit in the data: in a radiology report, a size of the spleen might be simply specified as ‘spleen 7 × 12 × 15 cm’ without any interpretation. Formalized medical knowledge can be used to classify this measurement finding as splenomegaly, i.e. an enlarged and thus abnormal spleen. Further, in a diagnostic process, finding data needs to be interpreted with respect to diseases. For example, a splenomegaly is an abnormal enlarged spleen and can be related to inflammation or to specific types of cancer such as lymphoma. Further, only lesions with a certain minimal size can be used as target lesions for treatment evaluation. In the following subsections, we show how the MCI is used to represent medical knowledge about the normal and abnormal size of anatomical entities, and how RECIST guidelines for target lesions can be formalized.

#### Normal size specifications

The medical literature contains much information about normal qualities of anatomical entities as well as descriptions of typical abnormal or pathological structures such as cysts, lesions or enlarged lymph nodes. The main function of these specifications is to define which manifestation of a quality of some anatomical entity is considered to be normal and which is not. Radiologists refer to these specifications from medical literature when they classify observed findings as normal, abnormal or pathological. For instance, a lymph node with a short axis of 0.7 cm is considered to be normal, since ‘normal lymph nodes are ≤ 1 cm in the short axis’. Accordingly, a lymph node of size 2.65 cm in the short axis is classified as abnormal. Similar specifications are available for many other anatomical entities (see e.g. [21–27]). The different types of specifications are listed in Table 1.

**Table 1:** Commonly used range specifications to describe the normal size of anatomical entities

Range type	Examples: anatomical entity E, quality Q, bound/range
Upper bound	lymph node (RID13296), short axis, 1 cm
	submental lymph node (RID7710), short axis, 1.5 cm
	spleen (RID86), length, 15 cm
Lower bound	ascending aorta (RID580), diameter root, 4 cm
Interval	kidney (RID205), cranio-caudal diameter, 8–13 cm
	wall of gallbladder (RID33779), thickness, 0.1–0.3 cm
	ureter (RID229), width, 0.4–0.7 cm

**Fig. 8:**
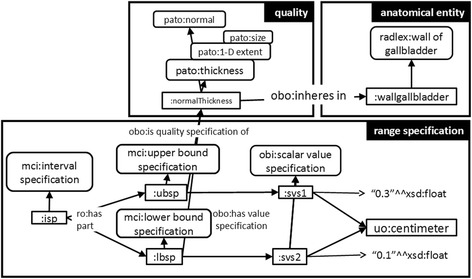
Normal size specification. The thickness of the wall of the gallbladder is normally in the range of 1–3 mm

The motivation for formalizing this knowledge is to automate this kind of classification in order to add information about the finding type. Thus, we enrich descriptions of clinical findings and enhance the data quality. As shown in Fig. 8, MCI defines classes for the different types of normal size specifications (i.e. interval, upper bound and lower bound). The pattern of these specifications is similar to the pattern of clinical findings described above.

The actual definition of which size of particular anatomical entities is considered to be normal depends on the guidelines used. However, we emphasize that the model is able to represent the necessary types of specifications (upper bound, lower bound and interval) and can be adapted to whichever guideline the user prefers. A representation of this type of medical knowledge is given in Fig. 8. In total, the knowledge model contains 40 size specifications related to 33 different anatomical entities. By using the RadLex subclass hierarchy, the overall coverage is enhanced: for instance, size descriptions about ‘lymph node’ apply to more than 250 subclasses in RadLex.

#### Normality classification of image findings

In [12], we demonstrated how structured representations of measurement findings can be extracted from free-text radiology reports by using Natural Language Processing technology in combination with the knowledge models described above. Here, we demonstrate how it is used to automatically classify image findings. First, we are given some size finding where the anatomical entity (E), the measured quality (Q) and the measured value are specified (as e.g. in Fig. 4). Then we check the knowledge model and retrieve the most specific size specification according to the Entity-Quality (EQ) methodology [28]. Only the normal size specifications with the most specific pair (anatomical entity E’, quality Q’) are retrieved. Most specific means that there is no size specification with anatomical entity E” and quality Q” such that E” is on the subclass path between E and E’, and Q” is on the subclass path between Q and Q’. If there are two such normal size specifications, the one with a shorter subclass path from E to E’ is considered the most specific. Thus, e.g., for a measurement of a submental lymph node, a different normal size specification is retrieved than for a measurement of a mediastinal lymph node. After retrieval, the normal range is compared to the actual measurement value. For example, the length of the spleen (9.6 cm) is compared to the normal length (7–10 cm), and accordingly the length is classified as normal and the finding as a normal finding. Since the classification of measurement findings is based on value comparisons, it is not realized by defining logical axioms in OWL. Instead, we use SPARQL to retrieve relevant data from the triple store and to classify findings by comparing their measurement values to normal size specifications of our knowledge model. The resulting finding type is then written back to the triple store.

### Response Evaluation Criteria In Solid Tumors (RECIST)

The RECIST (Response Evaluation Criteria In Solid Tumors) guideline allows a standardized assessment of tumor burden and tumor response during therapy. Here, a sum of the diameters (short axis for lymph nodes, longest diameter for the remaining lesions) for all target lesions is calculated and reported as the baseline for follow-up sum and indicates therapy response/failure. At baseline, tumor lesions (longest diameter ≥ 10 mm) and lymph nodes (short axis diameter ≥ 15 mm) can be considered and selected as target lesions. Up to a maximum of five lesions that are representative of all involved organs may be identified as target lesions (these should allow reproducible repeated measurements). The sum of the diameters of all target lesions is calculated and reported at baseline and during follow-up [3].

The following criteria are used to determine objective tumor response:

- Complete Response: Disappearance of all target lesions (any pathological lymph nodes, whether target or non-target, must have reduction in short axis to <10 mm).
- Partial Response: At least a 30 % decrease in the sum of diameters of target lesions (reference = baseline sum diameters).
- Progressive Disease: At least a 20 % increase in the sum of diameters of target lesions (reference = the smallest sum in the study; this includes the baseline sum if that is the smallest in the study). For Progressive Disease, the sum must also exhibit an absolute increase of ≥ 5 mm. The appearance of one or more new lesions is also considered as Progressive Disease.
- Stable Disease: Neither sufficient decrease to qualify for Complete/Partial Response nor sufficient increase to qualify for Progressive Disease [3].

Currently, automatic image segmentation algorithms are already used to detect lesions and lymph nodes in CT or MRI images (see e.g. [29–34]). Thus, automatic extraction of structured representations of lesion and lymph node size from images is within reach. With structured representations (e.g. in Fig. 7), large parts of the RECIST criteria can be automated and improved. Indeed, the guidelines represent rules and most of them are easily implemented using SPARQL:

1. Selection of target lesions: For each multidirectional measurement of a lymph node or lesion, the value for the short axis (longest axis respectively) is compared to the RECIST minimal value 15 mm (10 mm respectively). If the value is larger, then the finding about the lymph node (or lesion) is classified as a potential target lesion. The actual selection of final target lesions from the set of potential lesions is performed by the radiologist.
2. Verification of selected set of target lesions: It is verified that there are at most five target lesions selected and that each organ appears at most twice as the location of the lesion. This is realized by a SPARQL ASK query.
3. Calculation of RECIST sum: The values for short axis (lymph nodes) and longest axis (other lesions) are summed for all target lesions. The resulting value is represented as shown in Fig. 9.

**Fig. 9:**
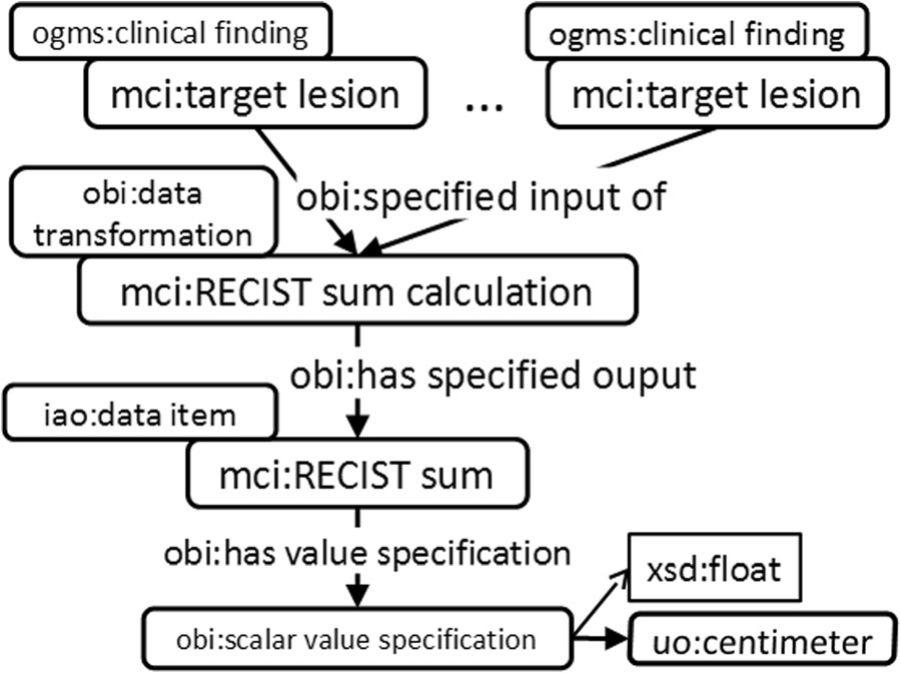
RECIST sum: this is calculated based on a set of target lesions. Adjacent items are represented by subclass relationships, i.e. the RECIST sum calculation is a subclass of data transformation
4. Classification of response: Classification of complete response and partial response is verified by SPARQL queries. Similarly, the increase for a progressive disease is verified. The appearance of new lesions has to be assigned manually by the radiologist. If not otherwise classified, the examination is assigned as a stable disease.

### ReportViewer

In addition to the classification of each finding as normal or abnormal, information about the change of findings over time is of importance. For instance, radiologists compare the size of lesions or specific anatomical entities such as lymph nodes or organs over time to evaluate the treatment success. For instance, the size of the spleen was measured in 3 dimensions in consecutive examinations and the radiologist ‘infers’ that the spleen size/volume increased, decreased or did not change. The comparison to the initial and the previously performed examination is required in many clinical processes such as differential diagnosis, monitoring or treatment evaluation. For instance, the radiologist has to examine which findings are progressive, regressive, stable or new.

Even though we have structured representations of findings that were extracted from text, automatic comparison requires the linking of consecutive findings. For findings about anatomical entities that can be identified only once in the human body (e.g. the liver or the spleen), this is possible. However, for lymph nodes and multiple lesions in one organ, the linking is not reliable: Two findings about a mediastinal lymph node from consecutive examinations do not necessarily describe the same entity. For lesions, the situation is similar: Two findings about a liver lesion from different reports do not necessarily describe the same lesion. Without a precise marking of the lesions by the radiologist, a direct and reliable link cannot be established.

**Fig. 10:**
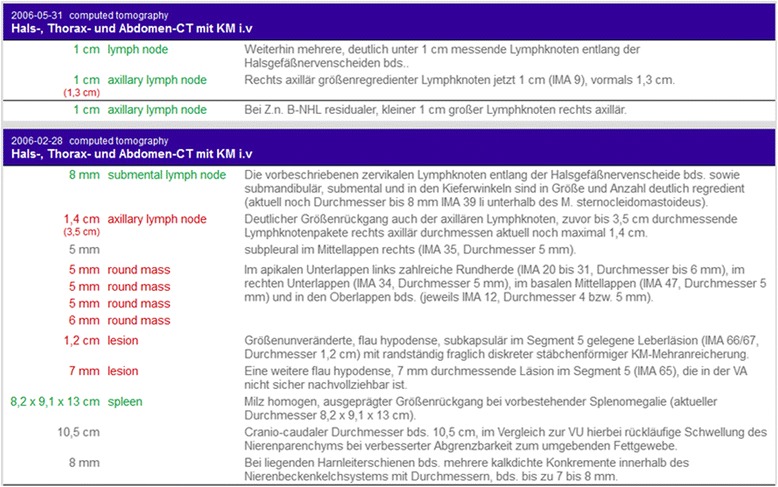
Screenshot of the ReportViewer showing extracted and classified measurement findings describing entities that are part of the thorax from consecutive examinations. The change of the size of the axillary lymph node from enlarged to normal is visible. *Red* = abnormal/pathologic finding, *green* = normal/non-pathologic finding

Thus, we decided to provide the radiologist with an integrated view of findings from consecutive examinations. The ReportViewer (Fig. 10) allows selective retrieval of findings from consecutive examinations. For instance, one can display findings about a particular anatomical entity (such as the spleen) or finding of a particular body region (thorax, abdomen, mediastinum, etc.) from consecutive examinations. Extracted and classified measurements are displayed next to the original report sentence. In total, the prototype implementation allows the radiologist to efficiently compare findings from consecutive examinations without losing the context information. As shown, e.g., in Fig. 10, the size of the axillary lymph node can be easily compared longitudinal (size changed from 3.5 to 1.4 to 1 cm). Thus the ReportViewer potentially facilitates follow-up examination.

## Results and discussion

In this section, we evaluate the normal classification and discuss related work.

### Evaluation of normal classification

#### Data sets

The evaluation is based on two data sets of radiology reports: Firstly, an oncologic data set was used consisting of 2584 German radiology reports (27 different readers) on 377 lymphoma patients. The imaging modality was mainly computed tomography (CT), but magnetic resonance imaging (MRI) and ultrasound (US) were also used. Secondly, a mixed data set consisting of 6007 German radiology reports (27 different readers) on diverse medical and surgical patients was used. The imaging modality was CT. The inspected body regions for both data sets were mainly abdomen, thorax and head, but also include various other regions from the whole body. Sentences with measurements were annotated with anatomical entities defined in RadLex, and the radiologist manually selected the best annotation to describe the measurement. For example, for the sentence ‘lymph nodes within aortopulmonary window and pretracheal unchanged with a size of up to 1.6 × 1.2 cm’ [Original in German: Die LK im aortopulmonalen Fenster und prätracheal größenunverändert mit einer Größe von bis zu 1,6 × 1,2 cm.], the pair (‘1.6 × 1.2 cm’, ‘aortopulmonary lymph node’) was selected by the radiologist. In total, 420/393 findings were classified in the oncologic/mixed data set (including 170/184 lesions and cysts).

#### Evaluation

All measurement findings, i.e. the pairs (measurement, anatomical entity), were automatically classified and for each data set, the radiologist was provided with an Excel spreadsheet that provided each classified finding, the sentence, the ‘measurement, anatomical entity’ pair and the classifier result (normal, abnormal or unclassified). Then the radiologist evaluated the classified finding as correct or incorrect. All unclassified findings were evaluated as incorrect. Accuracy was 96.4 % for oncologic reports and 92.9 % for mixed reports. Detailed evaluation results are summarized in Table 2. For both data sets, the number of incorrect classifications (i.e. false positives and false negatives) was very small (Table 2). However, both data sets have a significant number of unclassified findings that occur when the anatomical entities are not covered by the knowledge model.

**Table 2:** Evaluation results for the classification of normal and abnormal findings extracted from radiological reports

	Oncologic data set	Mixed data set
All cases	420	393
True normal	115	86
True abnormal	290	279
False normal	2	2
False abnormal	2	1
Unclassified	11	25
Accuracy	96.4 % (405/420)	92.9 % (365/393)

The oncologic data set contains reports from lymphoma patients; the mixed data set contains reports from diverse medical and surgical patients. ‘True normal’ means that the algorithm *correctly* classified a finding as normal (accordingly for ‘true abnormal’). ‘False normal’ means that the algorithm *falsely* classified a finding as normal while the radiologist classified it as abnormal (accordingly for ‘false normal’). The term ‘unclassified’ means that the algorithm was not able to classify the finding

Firstly, there are size findings about anatomical entities that are too unspecific to be classified as normal or abnormal. For instance, ‘portion of soft tissue’ is never classified for that reason (6 times for the oncologic data set and 9 times for the mixed data set). Similarly, ‘fluid’, ‘duct’ and ‘wall’ are not sufficiently specific for classification.

Secondly, there are anatomical entities that are simply not yet covered by the knowledge model. For instance, the finding ‘2.8 × 0.7 cm, rib’ is not classified because the knowledge model does not contain a normal size specification for the rib. Similarly, findings about pleura, trachea, right ventricle, bulla, infrarenal aorta, mucosa, L2 vertebral body, T10 vertebral body, L4 vertebral body, aortic arch, crus of diaphragm and set of biliary ducts were not classified. The rate of correctly classified findings for the oncologic data set is significantly higher than for the mixed data set, because fewer findings remained unclassified (11 out of 250). This is because the mixed data set contains reports from patients with diverse disease background and thus there are more measured anatomical entities that are not covered by the knowledge model. This can be resolved by extending the knowledge model for corresponding entities.

Most of the incorrect classifications are spleen measurements. For instance, the finding ‘14.8 × 8.5 cm, spleen’ was incorrectly classified as normal. This is because the classification algorithm simply maps 14.8 cm to the height (normal range: 11–15 cm) and the length (normal range: 7–10 cm). However, the size was measured in the axial plane and thus describes the length and width, and accordingly a splenomegaly, i.e. an abnormal spleen. On the other hand, the finding ‘9 × 3.5 × 6.5 cm, spleen’ was incorrectly classified as abnormal since the algorithm detected that the value 3.5 cm is below the normal width of 4–6 cm. However, according to the radiologist, this deviation is not considered to be abnormal. Here, the algorithm needs to be adapted accordingly. Further, the finding ‘4 cm, ascending aorta’ was incorrectly classified as normal. In this case, the size was compared to the normal size of the aorta at the root (normally at least 4 cm); however, the ascending aorta is normally slightly smaller, and thus 4 cm should have been classified as abnormal since it represents an ectasia of the ascending aorta. This fault can be avoided by extending the knowledge model with more granular size descriptions.

### Related work

In the biomedical domain, there is much work on structured representation of measurements from a wide variety of examinations. The contribution of our work is the integrated representation of image findings and medical knowledge. Here, we review existing modeling approaches.

We reused many different ontologies from the OBO library, and consequently patterns of these ontologies were reused. For example, the basic representation of a clinical finding is defined analogously to the representation of human phenotypes in the Human Phenotype Ontology (HP) [35, 36]. This has the advantage that clinical findings defined by MCI can be automatically classified in terms of HP such as splenomegaly, lymphadenopathy, etc. The pattern of scalar measurements is reused from the Ontology for Biomedical Investigations (OBI) [37].

In [38], the representation of phenotype measurement data is presented in which three aspects of a measurement are captured: the measurement itself, the method of how the measured values were obtained, and the condition of the measurement. While in laboratory tests these three aspects are of high relevance, for the radiology findings described here, the measurement method and condition (e.g. imaging modality or usage of a contrast agent) are of minor importance.

Another important related work is the Annotation and Image Markup (AIM) standard [39] that provides a data model for capturing image annotation and markup data. In particular, image measurements discussed in this paper are covered by AIM. Also, RadLex IDs are used as the controlled vocabulary for anatomical entities and observation characteristics. Thus, the approach and the knowledge model on normal size specifications could also be applied to image measurements specified in AIM, when qualities of measurements (length, width, thickness, etc.) are expressed at a similar level of granularity as PATO.

The Biological Spatial Ontology (BSPO) [40] is a small ontology specialized for cross-species representation of spatial anatomical entities. BSPO defines very useful classes for the representation of anatomical boundaries, planes and axes as well as many properties for spatial relations such as ‘anterior to’, ‘right of’, ‘on the distal side of’, etc. The problem, however, is that some classes and properties (with high relevance for our use case) were not fitting our need: Firstly, the ‘orthogonal to’ relation of BSPO is not symmetric and holds only between axes and planes - not between two axes within one plane. Secondly, the definition of the anterior-posterior axis does not match the human anatomy: In BSPO, the anterior-posterior axis is orthogonal to the axial plane and has the synonymous craniocaudal axis. For humans, however, the anterior-posterior axis is orthogonal to the coronal plane and different than the craniocaudal axis.

Thus, we decided to reuse classes for planes and axes from FMA instead and define orthogonal-to and parallel-to relations in MCI directly.

Biomarkers are measurable qualities of an organism that can be used to determine its status or condition (e.g. with respect to diseases or disorders). Since the structured representation of image measurements allows the analysis of large patient cohorts, MCI also supports the development of imaging biomarkers. Regarding this aspect, the Quantitative Imaging Biomarkers Ontology (QIBO) [41] was created to support the creation of biomarkers from clinical imaging data in general. The aim of QIBO is “to represent, integrate and harmonize heterogeneous knowledge across the domain of imaging biomarkers” [41]. However, QIBO is no longer maintained.

In radiology, structured reporting has been discussed for a long time. For example, in DICOM structured reporting (SR) [42], a hierarchical schema for the representation of image findings is presented that describes ‘Content Items’ by reference to some template and definition of properties such as the specification of a diameter or description of the measured entity. In contrast to DICOM SR, however, our representation is aligned with existing ontologies of the OBO library and thus automatically linked to further knowledge resources. In [43], the structure of DICOM SR findings and representations along UMLS semantic types is analyzed. Findings are distinguished as anatomical findings, lesion findings and quality findings. The goal is to link finding descriptions to diseases by using the GAMUTS ontology.

We mention that there are numerous Natural Language Processing (NLP) techniques to extract information from text. NLP techniques are employed by our approach for annotation of text with ontology concepts. In comparison to pure NLP approaches (such as [44]), however, our approach formalizes knowledge and thus is able to classify findings even when no interpretation such as ‘normal’, ‘unremarkable’, etc. occurs in the report text.

## Conclusions

In the biomedical domain, much work on the structured representation of measurements from a wide variety of examinations has been accomplished. The proposed MCI enables an accurate integrated representation of reported measurements and medical knowledge. Thus, measurements can be automatically classified and integrated in different clinical decision processes. The structured representation is suitable for better integration of clinical findings during decision-making. The proposed ReportViewer provides a longitudinal overview of the measurements. Further, MCI provides classes that are required for a RECIST-compliant representation of lymph node measurements and other lesions. Implementation of RECIST guidelines with SPARQL enhances the quality of the selection and comparison of target lesions, and the corresponding treatment response evaluation. In future work, we plan to contextualize the knowledge model with respect to the patient background such as age, gender, weight, height and other characteristics. Further, the integration with existing image segmentation algorithms will be a promising next step.
